# Primary hepatic neuroendocrine tumors: A case report

**DOI:** 10.1097/MD.0000000000018278

**Published:** 2019-12-16

**Authors:** Hai-Xia Hu, Tong Yu

**Affiliations:** a Department of MRI Room, Qinghai Provincial People's Hospital, Xining, Qinghai Province; b Department of Orthopedics, The Second Hospital of Jilin University, Changchun, Jilin Province, China.

**Keywords:** cirrhosis, hepatic, hepatitis, neuroendocrine, tumor

## Abstract

**Rationale:**

Primary hepatic neuroendocrine tumors (PHNET) are extremely rare, which makes it difficult for doctors not deeply to be aware of their imaging and pathological characteristics. Therefore, it is challenging to diagnose PHNET accurately without biopsy or surgical excision. The purpose of this study is

(1) to introduce the magnetic resonance imaging (MRI) and pathological characteristics of primary neuroendocrine tumors, so as to facilitate early diagnosis of PHNET to be made by doctors,
(2) and to introduce the treatment of PHNET.

**Patient concerns:**

A 52-year-old male patient came to our outpatient department with intermittent upper abdominal pain.

**Diagnoses:**

PHNET.

**Interventions:**

Biochemical examination and imaging examination were performed prior to operation. Liver tumors were removed by ultrasound scalpel under laparoscopy. Pathology examination of liver tumors was performed after operation. Symptomatic supportive treatment was performed after operation as well, including anti-inflammation and rehydration.

**Outcomes:**

The results of biochemical examination were generally normal. The results of MRI showed low signal on T1WI, slightly high signal on T2WI/FS and DWI manifestation of high signal. Immunohistochemistry (IHC) showed that synaptophysin (Syn) was positive, CD56 was positive, chromaffin A (CgA) was positive, and Ki-67 was 15%. The patient was generally in good condition and no discomfort or recurrence was reported during 15 months of follow-up.

**Lessons:**

The incidence of PHNET is extremely low. Sometimes the patient has no cirrhosis or hepatitis, and alpha-fetoprotein is not high, but imaging examination shows solid occupation and clear boundaries of the liver tumor, for which doctors should consider the primary liver nerve tumor. The diagnosis of PHNET depends on pathological characteristics. Surgical excision is the main method to treat the disease.

## 1 Introduction

Neuroendocrine tumors (NET) account for about 1% to 2% of all gastrointestinal tumor cases. However, PHNET is extremely rare.^[1–4]^ Edmonson et al^[5]^ reported PHNET for the first time. So far, the total number of reported cases worldwide is less than 150, accounting for about 0.3% of all neuroendocrine tumor cases.^[6]^ As these tumors account for only a small proportion of the whole NET, there is yet to be any formal diagnosis and treatment guidelines on the treatment of the disease, which often leads to the failure of early diagnosis and treatment for patients. Therefore, it is essential to expand the total database, which is conducive to the development of formal diagnosis and treatment guidelines, thereby improving the prognosis of patients. We report the diagnosis and therapeutic effect of a rare case of PHNET.

## 2 Ethical approval

The patient has given expressed consent to the publication of the case. This report was granted approval by the ethics committee of the Second Hospital of Jilin University, Changchun, China.

## 3 Case report

### 3.1 Patient characteristics

In April 2016, a 52-year-old male patient came to our outpatient department with intermittent upper abdominal pain. MRI revealed hepatic space-occupying lesions, but the patient rejected treatment and then left the hospital.

In April 2018, the above-mentioned symptoms quickly aggravated within a short period of time, and the re-examination by MRI revealed that the lesion was significantly larger than the previous one, which led to the diagnosis of liver space-occupying lesion.

### 3.2 Biochemical examination

Albumin was 38.4 g/L, glutamyl transferase was 112 U/L, serum amyloid A was 71.2 mg/L, and C-reactive protein was 20.5 mg/L. The results obtained from other tests such as tumor markers, blood test, coagulation and biochemistry test were generally normal.

### 3.3 Imaging examination

GE 3.0T superconducting MR scanner was applied. The scanning parameters are as follows.

(1) T2WI: TR 6000.0 ms, TE 85.00 ms, acquisition times 2.50, bandwidth 62.5 kHz, layer thickness 7.0 mm, layer spacing 1.0 mm, FOV 38 cm × 38 cm, and matrix 320 × 320;
(2) Three-dimensional LAVA: TR 2.80 ms, TE 1.40 ms, acquisition times 0.73, bandwidth 125.0 kHz, layer thickness 5.0 mm, FOV (34–38) cm × 34–38) cm, matrix 260 × 200; DWI: TR 2200.00 ms, TE 56.68 ms, acquisition times 2.00, bandwidth 250.0 kHz, layer thickness 7.0 mm, layer spacing 1.0 mm, FOV 38 cm × 38 cm, and matrix 128 × 160.

**Figure 1:**
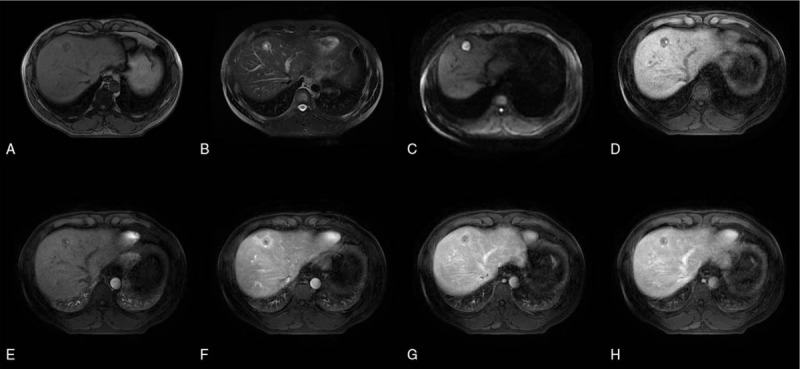
MRI was performed at initial admission. A 50-year-old male diagnosed as PHNET, grade G2. (A) Hybrid hypointense was observed on T1WI, and pseudocapsule could be seen. (B) Axis T2WI showed mixed high signal. (C) DWI image, (D) Enhanced anterior mask. (E) Early enhancement of arteries. (F) Late enhancement of arteries. (G) Enhanced venous phase. (H) Enhancement delay period.

**Figure 2:**
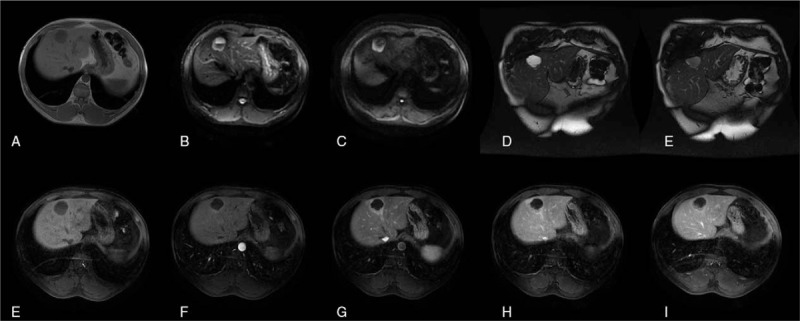
MRI results of 26 months after initial hospitalization showed that the diameter of the lesion increased from 40 mm to 60 mm. (A) Hybrid low signal on T1WI. (B) The axial T2WI lipid image showed mixed high signal. (C) DWI. (D) Coronal T2WI weighted images. (E) Coronal T2WI weighted images. (F) Enhanced anterior mask. (G) Early enhancement of arteries. (H) Late enhancement of arteries. (I) Enhanced venous phase. (J) Enhanced delayed phase.

The results of MRI are as follows (Figs. 1 and 2).

(1) Nodules and masses in the liver, about 6 × 5 cm in size, with clear boundary;
(2) Signal characteristics: low signal on T1WI and slightly high signal on T2WI/FS;
(3) DWI manifestations: high signal on DWI;
(4) cystic degeneration and necrosis: cystic degeneration and necrosis around the lesion;
(5) Pseudocapsule, dilatation of bile duct, hemorrhage and lipid signs around the lesion: pseudocapsule, dilatation of bile duct, hemorrhage, slightly decrease of flaky signal in inverse phase image around the lesion;
(6) Enhanced scan: enhancement in venous phase decreased, and inhomogeneity in arterial phase increased significantly.

### 3.4 Preliminary diagnosis

#### 3.4.1 PHNET

##### 3.4.1.1 Treatment method

Liver tumors were removed by ultrasound scalpel under laparoscopy. After sufficient hemostasis, a drainage tube was implanted. Pathology examination of liver tumors was performed. Postoperative symptomatic supportive treatment including anti-inflammation and rehydration was performed.

### 3.5 Pathological examination results

The resected pathological specimens showed wedge-shaped liver tissue with a size of about 7 × 3.5 × 5.5 cm. Cystic and solid masses were visible on the cut surface, which was gray-white and grey-red. In texture, central necrosis and hemorrhage were visible. The volume was 3 × 3.5 × 2.5 cm, the solid area was 3 × 2 × 1.5 cm, the cystic area was 1 to 1.8 cm, and the boundary was unclear.

**Figure 3:**
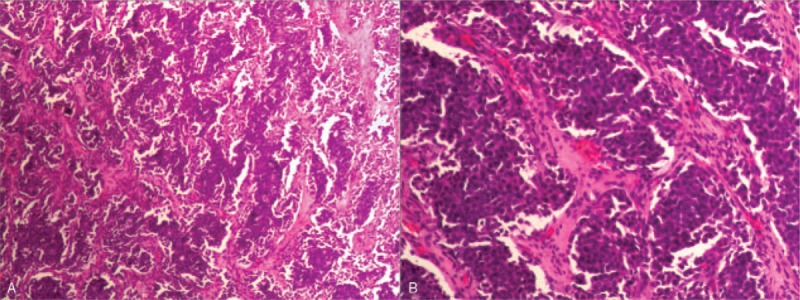
HE staining, (A) 4 × 10, (B) 10 × 10. Microscopically, the tumor cells showed diffuse distribution, infiltration growth, glandular tubular, spindle-shaped, local nest-like distribution, uniform cell size, darker staining, less visible nucleoli, increased mitotic image, accompanied by hemorrhage and necrosis.

HE staining (Fig. 3) showed that the tumor cells were spindle-shaped, glandular tubular, locally nested, aggregated, uniform in size, less visible nucleoli, deeper staining and increased mitotic image.

**Figure 4:**
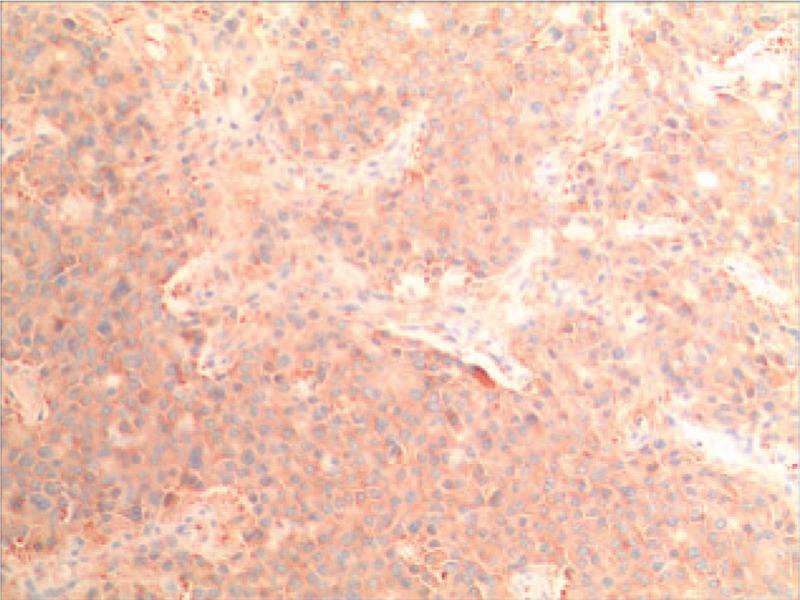
The results of IHC showed that the Syn was positive.

**Figure 5:**
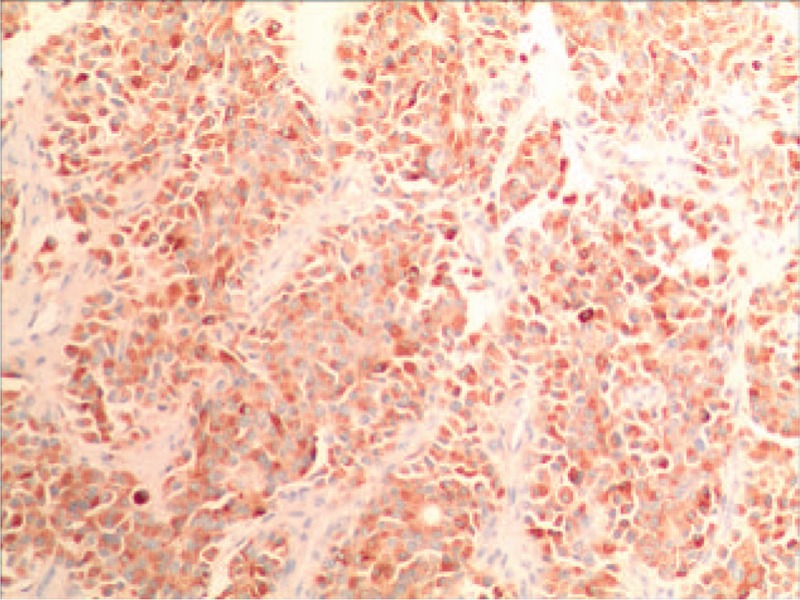
The outcomes of IHC explained that the CgA was positive.

**Figure 6:**
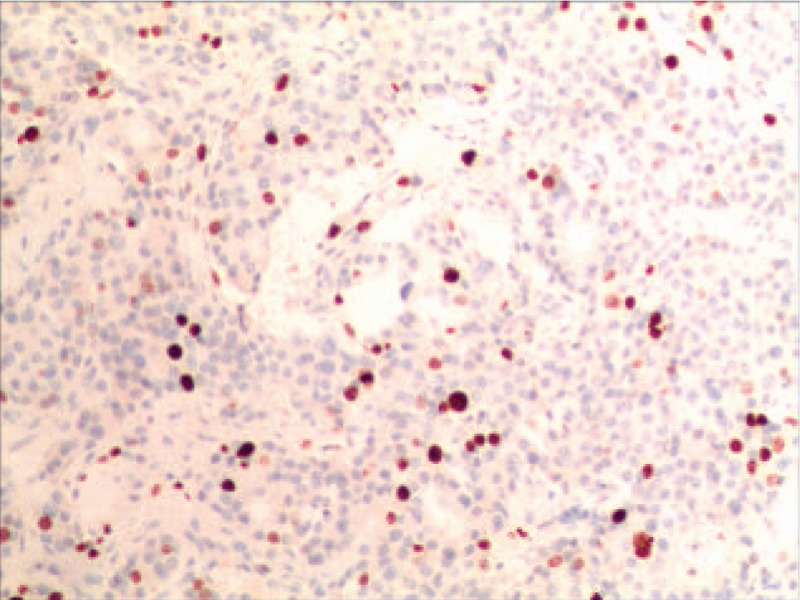
IHC demonstrated that the Ki-67 was 15%.

IHC indicated that Syn was positive (Fig. 4), CD56 was positive, CgA was positive (Fig. 5) and Ki-67 was 15% (Fig. 6).

### 3.6 Follow-up

No complications were found during 15 months follow-up visit. Moreover, the patient has a good quality of life postoperatively.

## 4 Discussion

NET is a kind of cancer with extremely low incidence. At present, its pathogenesis remains unclear. Scholars have proposed three hypotheses related to the origin of tumors. First, it stems from other ectopic tissues with endocrine function. Second, it is caused by the proliferation of neuroendocrine cells in the epithelium of intrahepatic capillary bile duct. Third, it stems from liver pluripotent stem cells.^[7]^ PHNET differs from other NETs as they do not produce biologically active polypeptides or amines, as a result of which there is no carcinoid syndrome manifested. In addition, PHNET grew slowly and most patients showed no symptoms in the early stage. When tumors were discovered, they had reached the middle or advanced stage, and the size of tumors was very large.^[1–3,8–10]^ In literature, it was reported that the size of tumors usually ranged from 0.4 cm to19 cm (mean 6.84 + 4.29 cm)^[11]^ when they were detected. Consequently, leading to delayed treatment.

### 4.1 Imaging characteristics

PHNET is highly similar to metastatic hepatitis NET, for which it is difficult to identify them.^[12]^ In order to accurately identify whether the primary lesions are located in extrahepatic, a adequate imaging examinations are recommended, including CT, MRI, enhanced MRI, PET and ultrasound.

Usually, the lesions exposed by CT scan show a low density. When the lesions become cystic, the lower-density areas can be observed. MRI showed long T1 and long T2 signals. If the lesions were in cystic degeneration, hemorrhage and fibrosis, they could show mixed signals. Usually, due to the high proportion of nuclear plasma, dense cell arrangement and less stroma, the diffusion of water molecules is inhibited. Therefore, the DWI sequence with significant effect on the examination of occult lesions is characterized by high signal intensity. In addition, the boundary of lesions in DWI sequence was better than those in conventional T1WI and T2WI.

The performance of PHNET enhanced scan is usually more complex. Some scholars^[13]^ conducted analysis of the dynamic enhanced CT manifestations in 38 cases of PHNET, of which 74% showed arterial phase enhancement, 52% exhibited delayed enhancement, and 48% showed hepatocellular carcinoma-like enhancement. Scholars^[14]^ hold the view that when the lesion is small (< 2 cm), it displays uniform enhancement. Conversely, when the lesion is large, it exhibits peripheral enhancement. In 2018, the use of indoleacetic acid-enhanced MRI was reported by Cha et al^[3]^ in an attempt to identify primary and metastatic NET.

PET/CT-guided biopsy, histopathology and IHC are conducive to the diagnosis of NET.^[15,16]^ PET/CT is incapable to directly diagnose PHNET, but it can be used to ascertain whether NET originates from liver.

### 4.2 Pathological manifestations

The final diagnosis of PHNET was premised on pathological findings. PHNET tissue specimens demonstrated a moderately hard mass, which is usually yellowish-brown on the section, with clear boundaries between the tumors and surrounding tissues, cystic lesions in the central area of the lesion, and dark red fluid in the lumen. Under the microscope, we can observe that the tumor cells have the same size, showing chrysanthemum, nest-like and strip-shaped structures. In some cases, abundant interstitial vessels and fibrous capsule can also be seen. The small and round nucleus is located in the middle of tumor cells, and has obvious heteromorphism.^[17]^ In this study, the volume of the tumors was 6 × 5 × 5 cm. The HE stained tumors were showed to contain glandular tubular and spindle-shaped cells with locally nested clusters. The cells were uniform in size, darker in staining and less visible in nucleoli. Our pathological results were found to be similar to those obtained by Song et al,^[18]^ who used HE staining for routine histopathological examination, which revealed nonspecific island, fusiform, nested, trabecular or mixed cell growth.

IHC markers include chromogranin A (CgA), neuron specific enolase (NSE) and synaptophysin (Syn). As suggested by the relevant studies, CgA and Syn are significant indicators to assist with the pathological diagnosis of PHNET.^[9]^ The optimal choice for diagnosis of PHNET is CgA, which is attributed to its sensitivity and specificity being much higher than other IHC markers. Besides, it has certain guiding effects on the prognosis and long-term monitoring of tumor patients. The positive expression of CgA and Syn in this case was found consistent with the results of IHC examination recorded in the literature.

Based on WHO digestive system classification,^[19]^ neuroendocrine tumors were classified into three categories, which are G1: mitotic < 2/10 HPF and/or PI < 2%; G2: mitotic < 2–20) / 10 HPF and/or PI < 3% to 20%; Neuroendocrine carcinoma (NEC): mitosis > 20/10 HPF and/or Ki-67 PI > 20%. There are studies^[20]^ demonstrating that mitosis, nuclear grading and Ki-67 PI are of great value for the evaluation of the malignancy and prognosis of PHNEN.

### 4.3 Clinical manifestations and treatment strategies

PHNET usually affects women aged 40 to 50 years.^[21–23]^ The most common clinical manifestations are abdominal distension and hepatic discomfort. In comparison, abdominal mass, weight loss, nausea, vomiting, and diarrhea are relatively rare.^[10]^ Tumors often overgrow, pressuring the patient's biliary system and leading to obstructive jaundice. A large proportion of the patients have no basic liver diseases, such as cirrhosis, hepatitis.^[6,24]^

Currently, surgery is preferred as the treatment of PHNET. Owning to the atypical clinical manifestations of PHNET, it is difficult to make diagnosis early. A majority of the patients have reached the middle or late stage when PHNET is found. Besides, the size of the tumors is large. Therefore, surgical resection is demanding in surgical skills.

As reported by Knox CD et al,^[23]^ the 5-year survival rate is as high as 78% under 70% hepatectomy. In this study, in order to extend the life span of patients, we performed tumors and partial hepatectomy. No complications were found during the 15-month period of follow-up. Chemotherapy ought to be performed after operation for small cell tumors. For PHNET patients who are unfit for surgical treatment, hepatic artery embolization and somatostatin conservative therapy may be worthy of consideration.^[25]^

## 5 Conclusion

The incidence of PHNET is extremely low. Sometimes the patient has no cirrhosis or hepatitis, and alpha-fetoprotein is not high, but imaging examination shows solid occupation and clear boundaries of the liver tumor, for which doctors should consider the primary liver nerve tumor. The diagnosis of PHNET depends on pathological characteristics. Surgical excision is the main method to treat the disease.

## Acknowledgments

We gratefully acknowledge the cooperation of the doctors and nurses in the operating room.

## Author contributions

**Methodology:** Hai-Xia Hu.

**Supervision:** Hai-Xia Hu.

**Writing – original draft:** Hai-Xia Hu, Tong Yu.

**Writing – review & editing:** Hai-Xia Hu, Tong Yu.
